# Indium-111-labeled CD166-targeted peptide as a potential nuclear imaging agent for detecting colorectal cancer stem-like cells in a xenograft mouse model

**DOI:** 10.1186/s13550-020-0597-3

**Published:** 2020-02-24

**Authors:** Siao-Syun Guan, Cheng-Tien Wu, Tse-Zung Liao, Tsai-Yueh Luo, Kun-Liang Lin, Shing-Hwa Liu

**Affiliations:** 1 Institute of Nuclear Energy Research, Atomic Energy Council, Taoyuan, Taiwan; 2 Department of Nutrition, China Medical University, Taichung, 40402 Taiwan; 3 Master Program of Food and Drug Safety, China Medical University, Taichung, 40402 Taiwan; 4 Institute of Toxicology, College of Medicine, National Taiwan University, No. 1, Jen-Ai Road, Section 1, Taipei, 10051 Taiwan; 5 Department of Medical Research, China Medical University Hospital, China Medical University, Taichung, Taiwan; 6 Department of Pediatrics, National Taiwan University Hospital, Taipei, Taiwan

**Keywords:** Cancer stem cells, Colorectal cancer, CD166-targeted peptides, Nuclear imaging agent probe

## Abstract

**Background:**

Cancer stem cells (CSCs) are involved in drug resistance, metastasis, and relapse of cancers, which can significantly affect tumor therapy. Hence, to develop specifically therapeutic target probe at CSCs for improvement of survival and quality of life of cancer patients is urgently needed. The CD166 protein has been suggested to be involved in colorectal cancer (CRC) tumorigenesis and to be considered a marker for colorectal CSCs (CRCSCs) detection. In this study, therefore, we attend to apply a nuclear imaging agent probe, Glycine_18_-Cystine-linked CD166-targeted peptides (CD166tp-G_18_C), to detect the changes of CD166 level in a CRC xenograft mouse model.

**Results:**

We isolated the CD166-positive cells from the HCT15 CRC cell line (CD166^+^HCT15) and evaluated their morphology and ability of clone formation, migration, protein expression, and drug resistance. The CD166-positive HCT15 cells display the CSCs characteristics. We discovered and designed a CD166-targeted peptide (CD166tp-G_18_C) as a targeted probe of CRC stem-like cell for cell binding assay. The CD166tp-G_18_C confirmed the CD166 protein targeting ability in CD166^+^HCT15 cells. The diethylenetriaminopentaacetic acid (DTPA)-conjugated CD166tp-G_18_C further was labeled with indium-111 (^111^In-DTPA-CD166tp-G_18_C) as nuclear imaging agent for imaging and bio-distribution analysis in vivo. Finally, we observed that the ^111^In-DTPA-CD166tp-G_18_C was significantly enhanced in tumor tissues of CD166^+^HCT15 xenograft mice as compared to the non-CD166tp-G_18_C control.

**Conclusions:**

Our results indicated that the indium-111-labeled CD166tp-G_18_C may be served as a powerful tool for colorectal CSCs nuclear imaging in the CRC patients.

## Background

Colorectal cancer (CRC) is the third most frequent occurring cancer in men and the second most frequent occurring cancer in women, with nearly 1.65 million new diagnosed cases and about 832,000 deaths in 2015 [1–3]. Although diagnostic techniques have improved dramatically, the rate of early diagnosis in CRC patients is still less than 40%. In the last decade, CRC has risen rapidly in Asia, and the cure rate has not improved remarkable, which leads to mortality increase and remains at about 12–14% in 5-year survival rate of patients with metastasis [4]. One possible cause of treatment failure is that the tumor site contains a small population of tumor-initiating cells termed cancer stem cells (CSCs). The CSCs presented high metastatic potential and contributed to chemoresistance and radioresistance through specific intracellular signal pathways, such as Wnt, Notch, and Hedgehogand [5]. The colorectal cancer stem cells (CRCSCs) had been identified in human CRC tumor tissues [6, 7] and were considered to be the crucial targets for the effective treatment of CRC [8]. Several studies showed that CRCSCs-targeted therapy effectively reduced the chance of tumor recurrence and metastasis [9–11]. The accurately diagnostic tool contributes to the efficacy in assessment of CRCSCs maintenance and metastatic sites. Colonoscopy is a most versatile diagnostic technique in symptomatic individuals including colon cancer [12]. However, the CSCs in the tumor tissue biopsy are not easily removed by colonoscopy. The small population of CSCs may affect the results of biopsy and interpretation of subsequent analysis. Therefore, development of non-invasive nuclear medicine imaging with high specific biomarkers is suitable as a tool for CRCSCs detection.

CD166, also known as activated leukocyte cell adhesion molecule (ALCAM), is a member of the immunoglobulin superfamily of cell adhesion molecules for intercellular adhesion regulation [13, 14]. It is a highly conserved 110-kDa type-I transmembrane glycoprotein, which comprises five extracellular Ig domains: two amino-terminal variable (V) type Ig domains followed by three constant (C) type Ig domains (V1V2C1C2C3), a lipophilic transmembrane domain, and a short cytoplasmic tail [15]. CD166 has been reported to be involved in many biological activities, such as T cell activation and proliferation, monocyte migration, axon fasciculation, angiogenesis, and hematopoiesis [16, 17], and has been suggested to be correlated with aggressive disease phenotype in many cancers including CRC [18], esophageal cancer [19], melanoma [20], breast [21], ovarian [22], and prostate [23]. It is also one of the biomarkers associated with CRCSCs, which are responsible for CRC tumor initiation in xenografts, colony formation, and further enrichment [24, 25]. In addition, CD166 also contributed to the survival time and cancer regression of patients with CRC in which the frequently upregulated CD166 can act as an independent prognostic marker in the progression of CRC [26, 27]. These findings indicated that CD166 is a key factor for maintaining the characteristics of CRCSCs and possesses the potential as a prognostic biomarker in the clinical management of CRC patients. Therefore, development of a highly specific CD166-targeted probe for estimating CD166 status is helpful for the optimal care in patients with advanced CRC.

Antibody-drug conjugates (ADC) have been widely used as the specific biomarker ligands for tumor targeting in clinical and preclinical studies [28, 29]. However, the macromolecule antibodies might lead to an immune response, low tumor penetration, high accumulation in specific tissues/organs, and resistance [30]. Therefore, in order to overcome ADC limitations, the development of new specific targeting peptide ligands as imaging probe is a more effective strategy [31]. To date, the phage-displayed peptide library is a potential tool for identifying specific peptide ligands. The method has been applied in lymphocyte epitope mapping, selection of bioactive peptides bound to receptors/proteins, and development of drug delivery systems [32]. Recent studies have reported that the peptide selection method successfully discovered novel peptides as imaging probe for tumor detection [33, 34].

In this study, we aimed to design an effective CD166-targeted peptide as a nuclear imaging probe for CRCSCs detection. We developed an optimal CD166-targeted peptide (CD166tp) by using phage-displayed peptide library, which linked with Glycine_18_-Cysteine (G_18_C), and conjugated with diethylenetriaminopentaacetic acid (DTPA) for isotope (Indium-111) labeling. We used this CD166-targeted radiopharmaceutical for CD166 detection in a CRC xenograft mouse model.

## Materials and methods

### Reagents

Fluorescein isothiocyanate isomer I (FITC) and cyanine5.5 NHS ester (Cy5.5) were obtained from Sigma-Aldrich (St. Louis, MO, USA). CD166-targeted peptides (CD166tp, amino acid sequence: DSEGNSNLCSQS), CD166tp-C (amino acid sequence: DSEGNSNLCSQSC), G_18_C (amino acid sequence: GGGGGGGGGGGGGGGGGGC), CD166tp-G_18_C (amino acid sequence: DSEGNSNLCSQSGGGGGGGGGGGGGGGGGGC), FITC-labeled CD166tp-G_18_C (CD166tp-G_18_C-FITC), G_18_C-FITC, IgG-FITC, and FITC-conjugated CD166 antibody (CD166ab-FITC) were customizedly synthesized by Sigma-Aldrich. The Maleimide-DTPA was obtained from CheMatech (Dijon, Bourgogne-Franche-Comté, France).

### Cell culture

The human CRC-derived cell line (HCT15) was obtained from the Bioresource Collection and Research Center (Hsinchu, Taiwan). Cells were cultured in RPMI-1640 medium (Thermo Fisher Scientific, Waltham, MA, USA) with 10% fetal bovine serum (FBS) at 37 °C in a humid incubator with 5% CO_2_.

### Animals

The 10-week-old male BALB/c nude mice were obtained from BioLASCO (Taipei, Taiwan) and housed under the standard condition (a 12:12-h light:dark cycle at 22 ± 2 °C). Mice were supplied rodent laboratory chow diet and drinking water ad libitum. Animal protocols were approved (INER-107175) by the institutive ethical review committee and were in accordance with regulations in Taiwan and National Institute of Health guidelines on the care and welfare of laboratory animals.

### Magnetic-activated cell sorting of CD166 cells

HCT15 cells were treated with type I collagenase, and then the digested cells were collected and passed through a 40-μm nylon cell strainers (1 × 10^7^ cells/mL). These cells were suspended in PBS and FcR blocking buffer and then added anti-CD166 human polyclonal immune-magnetic beads (Thermo Fisher Scientific). After washing bead-bound cells, the CD166-positive HCT15 cells (CD166^+^HCT15) were obtained. The purity of both CD166^+^HCT15 and CD166-negative HCT15 cells (CD166^−^HCT15) was detected using standard flow cytometric analysis (see below).

### Screening of CD166-targeted peptides by phage display

The Ph.D.-12™ Phage Display Peptide Library Kit (New England Biolabs, Ipswich, MA, USA) was used for screening human CD166-targeted peptides according to the manual protocol of manufacturer. Briefly, the 20 μg human CD166 recombinant protein (Sigma-Aldrich) was coated on 96-well plate with 100 μL NaHCO_3_ solution (0.1 M, pH 8.6) overnight at 4 °C. Next day, each well was blocked with 250 μL blocking buffer (0.1 M NaHCO_3_, pH 8.6, 5 mg/mL BSA) for 2 h at room temperature (RT). All wells were washed 6 times with TBST (0.1% Tween-20, pH 7.5) for biopanning. For the phage selection procedure, each well was added with phage library (10^11^ plaque forming unit (pfu)/100 μL in TBST with 0.1% Tween-20) and incubated for 1 h at RT. After washing 10 times with TBST (0.1% Tween-20), the bound phages were eluted with 150 μL elution buffer (0.2 M Glycine-HCl, pH 2.2, 1 mg/mL BSA) and neutralized immediately with 15 μL neutralizing buffer (1 M Tris-HCl, pH 9.1). Five microliters of eluted phages was then used for tittering. The remaining phages were amplified for subsequent rounds of biopanning (second to fourth rounds) that the detergents at the concentrations of 0.3% (second round), 0.5% (third round), and 0.5% (fourth round) were used. For the amplification procedure, the eluted phages were infected in chemically competent E. coli cells (ER2738) for 5 h at 37 °C. After bacteriolysis, the phages were recovered from the culture supernatant by using centrifugation and PEG precipitation. The titer was determined by a blue/white colony screening on LB/IPTG/Xgal plate and used as input phages for the next cycle of biopanning. The phage recovery rate of each round was calculated as follows: titer of output phage/titer of input phage × 100%. After the final round of biopanning, the phage clones with higher binding affinity to CD166 protein were collected for DNA extraction. To the amplified phages, 100 μL were added iodide buffer (10 mM Tris-HCl pH 8.0, 0.1 mM EDTA, 4 M NaCl) by vigorously tapping the tube and added 250 μL 95% ethanol for 20 min at RT. After centrifugation, the pellets were washed with 0.5 mL 75% ethanol and briefly dried under vacuum. Finally, the pellets were resuspended in ddH_2_O for DNA sequencing. The resulting sequences were analyzed to identify homologous amino acid sequences (Table 1). To further select the best targeted peptide sequence, the phage clones (clone number 1, 2, 3, 4, 5, 7, 10, 11) corresponding to sequence peptide (P1–P8) were chosen for phage ELISA assay and cell-based phage ELISA assay.

**Table 1:** Properties of peptides displayed by specific CD166-binding phages

Peptide	Phage clones	Sequences	MW	pI
P1	1, 8, 19, 22, 25	EGHCNNQICSNQ	1346.42	5.1
P2	2, 14, 15, 29	ETETQGTVCGGC	1184.27	3.1
P3	3, 6, 13	KCDNGTVACNQT	1253.38	5.9
P4	4, 18, 24, 28	DNKCSNTAQTNG	1252.28	6.1
P5	5	HQHGNNTIGGNS	1235.24	8.0
P6	7, 9, 12, 16, 21, 23, 27, 30	DSEGNSNLCSQS	1240.23	3.0
P7	10, 26	KGRPQSTMPNSQ	1330.49	11.5
P8	11, 17, 20	DNESSTNITQTS	1296.27	3.0

*MW* molecular weight, *pI* isoelectric point

### Phage ELISA assay

The 96-well plates were coated with 150 μL (50 μg/mL) human CD166 recombinant protein and BSA (as a control) in 0.1 M NaHCO_3_ (pH 8.6) overnight at 4 °C. After blocking with 250 μL blocking buffer (0.1 M NaHCO_3_, pH 8.6, 5 mg/mL BSA) for 2 h at RT, the final round of eluted phage clones (nos. 1, 2, 3, 4, 5, 7, 10, 11) were amplified and 100 μL 10^11^ phages diluents were added to each well and incubated at 37 °C for 2 h. After washing the plate for 6 times with TBST (0.5% Tween-20), 100 μL of HRP-conjugated M13-monoclone antibody (1:5000; Abcam, Cambridge, UK) was added and the plate was incubated for 2 h at RT. The mixture of chemiluminescent substrates (150 μL/well) was then added to the wells for reacting 10 min. The reaction was stopped with 2 M sulfuric acid (50 μL/well). The absorbance of each well at 450 nm was detected with an ELISA reader (Wallac 1420 VICTOR2™; Perkin Elmer, Waltham, MA, USA).

### Cell-based phage ELISA

Both CD166^+^HCT15 and CD166^−^HCT15 cells were used to evaluate the binding of selected phage clones on cell surface. Both cell lines were cultured in 96-well plates to 80% confluence and fixed with 4% paraformaldehyde. After blocking with BSA (5 mg/mL) for 2 h at RT, 10^11^ individual phages were added to each well and incubated at 37 °C for 2 h. After washing the plate with PBST for 6 times, the cell-bound phages were detected with HRP-conjugated M13-monoclone antibody (1:5000; Abcam) as described above.

### Flow cytometry analysis

For CD166 detection on the cellular surface, the optimized density (1 × 10^6^ cell) of CD166^+^HCT15 and CD166^−^HCT15 cells were added with 1 mL PBS with 20 μg IgG-FITC and FITC-conjugated CD166 antibody (CD166ab-FITC) for 1 h. For the CD166tp-G_18_C binding assay, CD166^+^HCT15 and CD166^−^HCT15 cells were added with 1 mL PBS with 20 μg CD166tp-G_18_C-FITC and G_18_C-FITC for 1 h. In competitive group, CD166^+^HCT15 cells were pre-treated with CD166tp-G_18_C (20 μg/mL) for 1 h and then added 20 μg/mL CD166tp-G_18_C-FITC for 1 h. After PBS washing, cells were collected for flow cytometric analysis using a FACSCalibur Flow Cytometer (BD Bioscience, San Diego, CA, USA).

### Immunoblotting

The samples were loaded in a 10% SDS polyacrylamide gel electrophoresis (SDS-PAGE), and then the proteins were transferred to polyvinylidene difluoride membranes (Bio-Rad; Hercules, CA, USA). After blocking 30 min at 4 °C (blocking reagent, Goal Bio, Taipei, Taiwan), the membranes were then incubated with primary antibodies against CD166 (1:2000) (Sigma-Aldrich), Nanog (1:1000), c-Myc (1:1000), OCT4 (1:2000), and Survivin (1:2000) (Cell signaling technology; Danvers, MA, USA) at 4 °C overnight. After washing procedure, membranes were incubated with secondary antibody (1:3000) (Sigma-Aldrich) at 4 °C for 1 h. Finally, the membranes were covered with enhance chemiluminescence substrate (Thermo Fisher Scientific) for 1 min and analyzed by using a luminescent image analyzer (LAS-4000 mini; GE Healthcare, Uppsala, Sweden). Band densitometry was quantified by Multi Gauge v3.2 software (GE Healthcare).

### Tumor sphere assay

Both CD166^+^HCT15 and CD166^−^HCT15 cells (at a density of 1 × 10^4^ cells/well) were cultured in 6-well ultra-low attachment plates with MSC Nutristem® XF medium (Biological industries, Cromwell, CT, USA) without FBS. After 10 days, the spherical cells (> 50 μm) were counted by using a microscope.

### Clone formation experiment

Both CD166^+^ and CD166^−^ HCT15 cells were separated into single cells (2000 cells/well) and plated into culture dishes (diameter, 6 cm) to grow for 16 days. The medium (MSC Nutristem® XF medium supplemented without FBS) was replaced every 3 days. The cell colonies were fixed with 10% neutral buffered formalin solution for 30 min and stained with 0.05% (g/L) crystal violet solution for 30 min.

### Migration assay

The cells with 90% confluence in the six-well plate were gently created a horizontal wound in monolayers using a 200-μL sterile pipette tip. The scratch images were acquired at × 100 magnification at 0 h (T0) and 24 h (T24). The migration distance was determined by using ImageJ software to detect the reduction of the wound gap.

### Cell viability assay

The cellular viability was determined by a cell counting kit-8 (CCK-8) kit (Sigma-Aldrich). For cell resistance assay, CD166^+^HCT15 and CD166^−^HCT15 cells were treated with Doxorubicin and 5-fluorouracil (1, 5, and 50 μM) for 24 h. The absorbance (450 nm) was detected by a microplate reader (Bio-Rad).

### Immunofluorescence staining

The CD166tp-G_18_C binding assay was determined by immunofluorescence staining in both CD166^−^HCT15 and CD166^+^HCT15 cells. Briefly, cells (1 × 10^4^ cells) were seeded in 4-well Millicell EZ slides (Merck Millipore, Germany) with culture medium at 37 °C and 5% CO_2_ for 16 h. Cells were fixed using 4% paraformaldehyde for 30 min at 37 °C. The fixed-cells were then incubated in the SuperBlock Blocking Buffers (Thermo Fisher Scientific, USA) for 30 min. Cells were incubated with CD166tp-G_18_C-FITC (20 μg/mL) for 2 h. DAPI was used to stain the cell nuclei at a concentration of 0.2 μg DAPI/mL PBS for 10 min. The images were captured by a fluorescence microscope (BX53, Olympus, Japan) coupled to a CCD camera. Digital images were analyzed by an Image-Pro Plus software (Media Cybernetics, USA).

### CD166tp-G_18_C and CD166 protein interaction assay

HCT15 cells (1 × 10^6^ cells) were incubated with 20 μg/mL CD166tp-G_18_C-FITC in the presence or absence of 20 μg/mL CD166tp-G_18_C at 4 °C for 1 h. After treating immunoblotting lysis buffer with protease inhibitors cocktail (Hycell International, Taipei, Taiwan), the mixture solution was centrifuged at 14,000 rpm (4 °C) for 10 min. The supernatant was treated with or without 2 μg/mL biotinylated-CD166 antibody (Novus Biologicals, Littleton, CO, USA)-conjugated streptavidin agarose beads (Sigma-Aldrich) at 4 °C overnight. In the competitive group, cells were pre-treated with CD166tp-G_18_C (20 μg/mL) for 1 h at 4 °C. The fluorescence of immunoprecipitates was detected by an ELISA reader (Perkin Elmer).

### CD166 fluorescence imaging and distribution in CRC xenograft mice

Both CD166^+^HCT15 cells and CD166^−^HCT15 cells were collected for subcutaneously inoculating into the flank of nude mice for 2 weeks. For the CD166 imaging assay, the Cy5.5-labeled CD166 antibody was intravenously injected into tail vein of both CD166^+^HCT15 and CD166^−^HCT15 xenograft mice. The fluorescent images of whole animal body were detected by an in vivo fluorescent imaging system (Perkin Elmer). Mice were sacrificed and the organs (brain, heart, lung, liver, spleen, kidneys, stomach, small intestine, colon, muscle, and tumor) were collected for fluorescent distribution imaging detection.

### Preparation of DTPA-conjugated CD166tp-G_18_C

For synthesis of DTPA-conjugated CD166tp-G_18_C (DTPA-CD166tp-G_18_C), the CD166tp-G_18_C and maleimide-DTPA (w/w 1:50) were dissolved in sodium carbonate buffer (pH 8.0) for 8 h, and then the reacted mixture was purified by HPLC (Ultimate 3000; Thermo Dionex, Sunnyvale, CA, USA) on a C18 column using a linear gradient of 10 to 80% acetonitrile/water (0.1% trifluoroacetic acid) for 30 min at a flow rate of 0.3 mL/min. The molecular weights of maleimide-DTPA, CD166tp-G_18_C, and DTPA-CD166tp-G_18_C were detected by a matrix-assisted laser desorption/ionization time-of-flight mass spectrometry (ultraflexIII MS TOFTOF; Bruker Daltonics, Billerica, MA, USA). The spectra were processed by a FlexAnalysis™ 3.0 software (Bruker Daltonics). For procedure of DTPA-CD166tp-G_18_C labeled with indium-111, 0.1 mg DTPA-CD166tp-G_18_C and 370 MBq Indium-111 were reacted in PBS buffer (pH 7.4) at RT for 1 h. Then, the labeling efficiency of ^111^In-DTPA-CD166tp-G_18_C was detected by instant thin-layer chromatography (ITLC). Briefly, the labeling solution was dripped on silica gel-impregnated glass fiber sheets (PALL Corporation, USA) using PBS buffer, pH 7.4, as the mobile phase. After sheets were dried with a heater, the radio-TLC Imaging Scanner (AR-2000, Bioscan, Washington, DC, USA) was performed for detected the radiation signaling.

### The stability assay of ^111^In-DTPA-CD166tp-G_18_C *in vitro*

For ^111^In-DTPA-CD166tp-G_18_C stability assay, the 18.5 MBq ^111^In-labeled compound was incubated with human, fetal bovine, and mouse serum in a 6-cm dish at 37 °C for 144 h, respectively. The radio-labeling yields of ^111^In-DTPA-CD166tp-G_18_C were detected by ITLC on silica gel-impregnated glass fiber sheets (PALL corporation, USA) using PBS buffer, pH 7.4, as the mobile phase every 24 h.

### The CD166 nuclear imaging in a CRC xenograft mouse model

The male BALB/c nude mice were subcutaneously inoculating CD166^+^HCT15 cells (1 × 10^6^ cells) for 2 weeks, and then the ^111^In-DTPA, ^111^In-DTPA-G_18_C, ^111^In-DTPA-CD166tp-C, and ^111^In-DTPA-CD166tp-G_18_C (740 MBq/kg/mouse) were intravenously injected into mice. The imaging of CD166 in mice at 2, 4, 24, and 48 h were detected by a nano single-photon emission computed tomography/computed tomography (nanoSPECT/CT; Mediso Medical Imaging Systems, Budapest, Hungary). For competitive study, the CD166^+^HCT15-derived xenograft mice were pre-treated with CD166tp-G_18_C (0, 10, and 50 mg/kg) for 6 h. Every mouse then received 740 MBq/kg ^111^In-DTPA-CD166tp-G_18_C via intravenous injection for 24 and 48 h. The competitive CD166 images were observed by a nanoSPECT/CT.

### Ex vivo bio-distribution study

CD166^+^HCT15 cells were subcutaneously injected into the right hindlimb of mice for establishing CD166-expressed CRC xenograft mouse model. After tumor formation, the ^111^In-DTPA, ^111^In-DTPA-G_18_C, ^111^In-DTPA-CD166tp-C, and ^111^In-DTPA-CD166tp-G_18_C (148 MBq/kg/mouse) were intravenously injected into mice for 2 weeks. After 2, 4, 24, and 48 h post-injection, mice were sacrificed and collected blood and organs including the brain, heart, lung, liver, spleen, kidneys, stomach, small intestine, colon, muscle, and tumor. The organ weight was measured. The radioactivity in blood and organs was determined by a 1470 WIZARD gamma counter (PerkinElmer). The percentage of injected dose per gram of tissue (%ID/g) was calculated.

### Statistical analysis

Data were presented as mean ± standard deviation. Groups more than two were determined by one-way analysis of variance (ANOVA) followed by post hoc analysis with Bonferroni’s test. The significant differences (*p* value < 0.05) among experimental groups were analyzed by GraphPad Prism V5.01 software (San Diego, CA, USA).

## Results

### CD166^+^ cells have characteristics of CSCs and present drug resistance

**Fig. 1:**
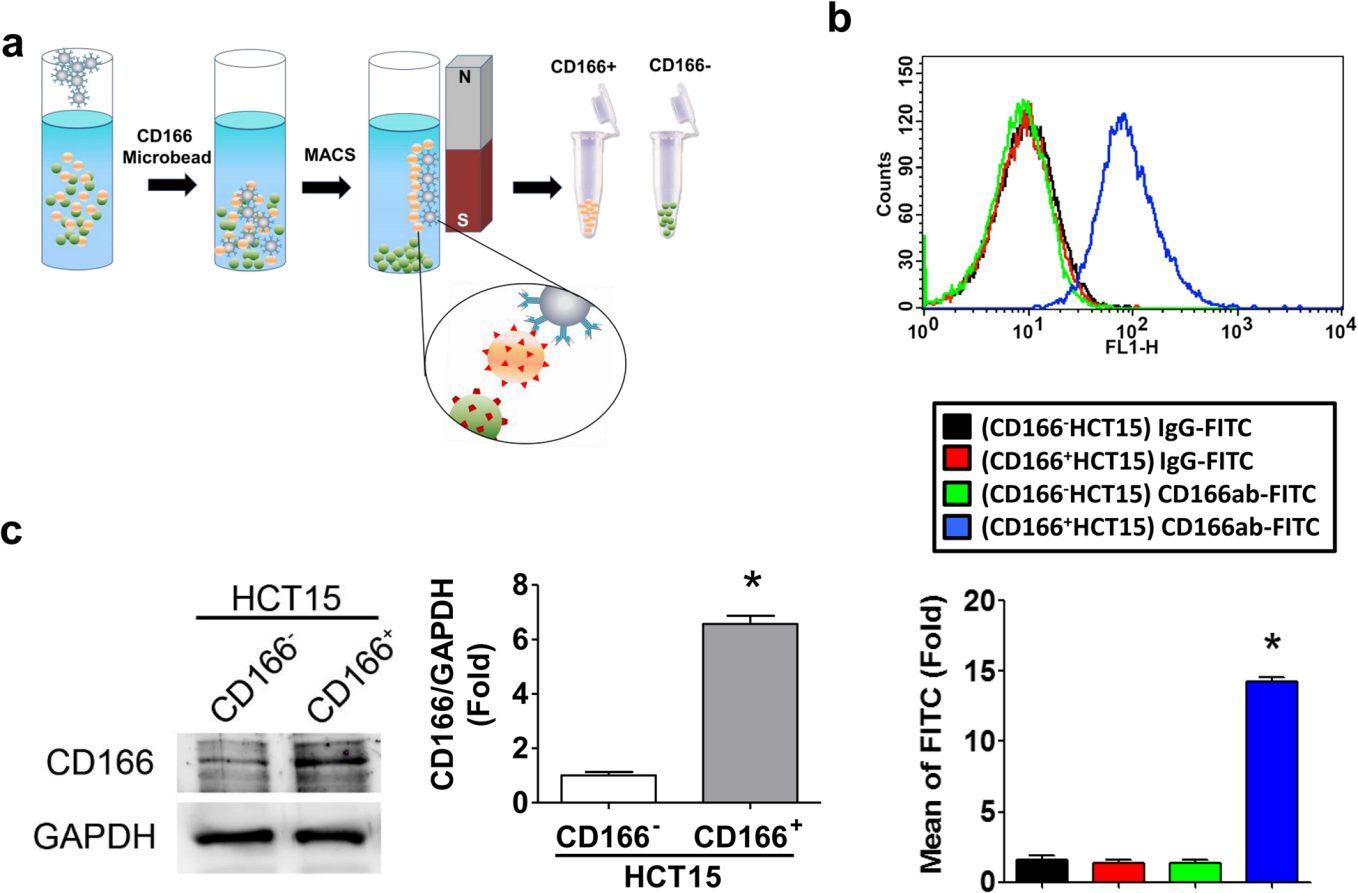
Isolation and analysis of CD166^+^HCT15 cells. **a** Schematic diagram for the isolation of CD166^+^HCT15 cells by magnetic-activated cell sorting (MACS). **b** The rate of CD166 expression in HCT15 cell sorted population determined by flow cytometry. Both CD166^+^HCT15 and CD166^−^HCT15 cells were treated with IgG-FITC and FITC-labeled CD166 antibody (CD166ab-FITC, 20 μg/mL) for 1 h. Data are presented as mean ± SD (*n* ≥ 3). *P < 0.05, versus IgG-FITC. **c** The protein levels of CD166 in both CD166^+^HCT15 and CD166^−^HCT15 cells. The protein expression was detected by Western blotting and quantified by densitometry and normalized by GAPDH levels. The data are presented as mean ± SD (*n* ≥ 3). *P < 0.05, versus CD166^−^HCT15

The CD166 antibody-conjugated magnetic beads were used to isolate the CD166^+^HCT15 cells (Fig. 1a). After the immunomagnetic sorting with CD166 beads, the CD166-expressed cells were determined by Western blotting and cytometry. The results indicated that the CD166^+^HCT15 and CD166^−^HCT15 cells could be separated by magnetic beads (Fig. 1b, c).

**Fig. 2:**
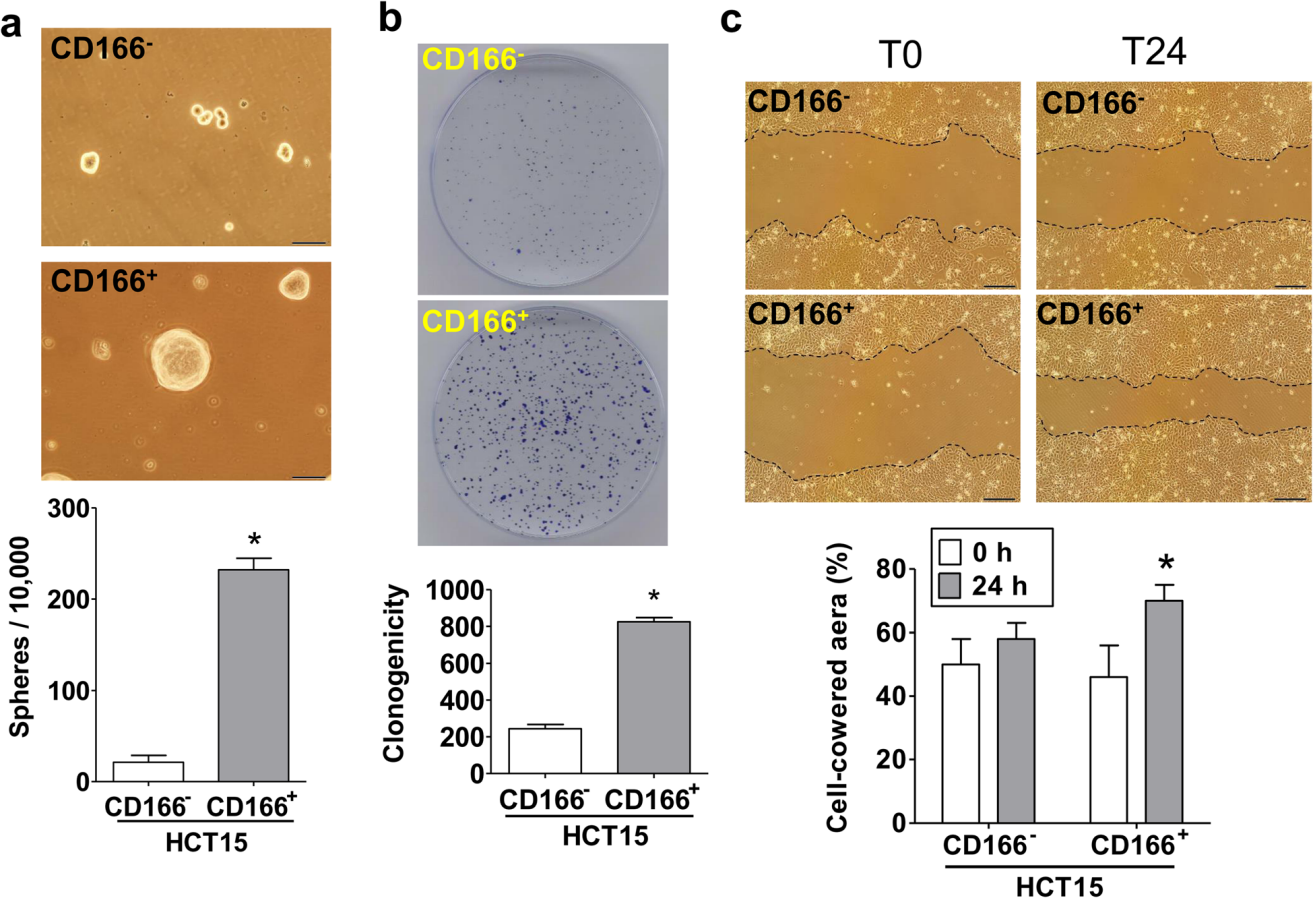
Identification of cancer stem cell feature in CD166^+^HCT15 cells. **a** Morphological characteristics during the growth of tumor spheres for 14 days. The quantitation of cell sphere formation was detected by manual calculation under light microscopy. Magnification, × 400; scale bar, 50 μm. **b** Colony formation of CD166^+^HCT15 and CD166^−^HCT15 cells. The representative image was shown and the colony number was quantified. **c** Cell migration assay. A wound was made in the monolayer in both CD166^+^HCT15 and CD166^−^HCT15 cells (T0), and then the cells were allowed to migrate for 24 h (T24). The quantification of wound closure was determined by ImageJ software. Magnification, ×100; scale bar, 200 μm. In **a**–**c**, data are presented as mean ± SD (*n* = 3).*P < 0.05, versus CD166^−^ group

CD166 was considered a biomarker for colorectal cancer stem cells (CSCs). We next tested the features of CSCs in these CD166^+^ cells using the in vitro sphere formation assay, clone formation assay, and migration assay. CD166^+^HCT15 cells presented spheroids and higher colony-forming efficiency, and migration ability than that of CD166^−^HCT15 cells (Fig. 2).

**Fig. 3:**
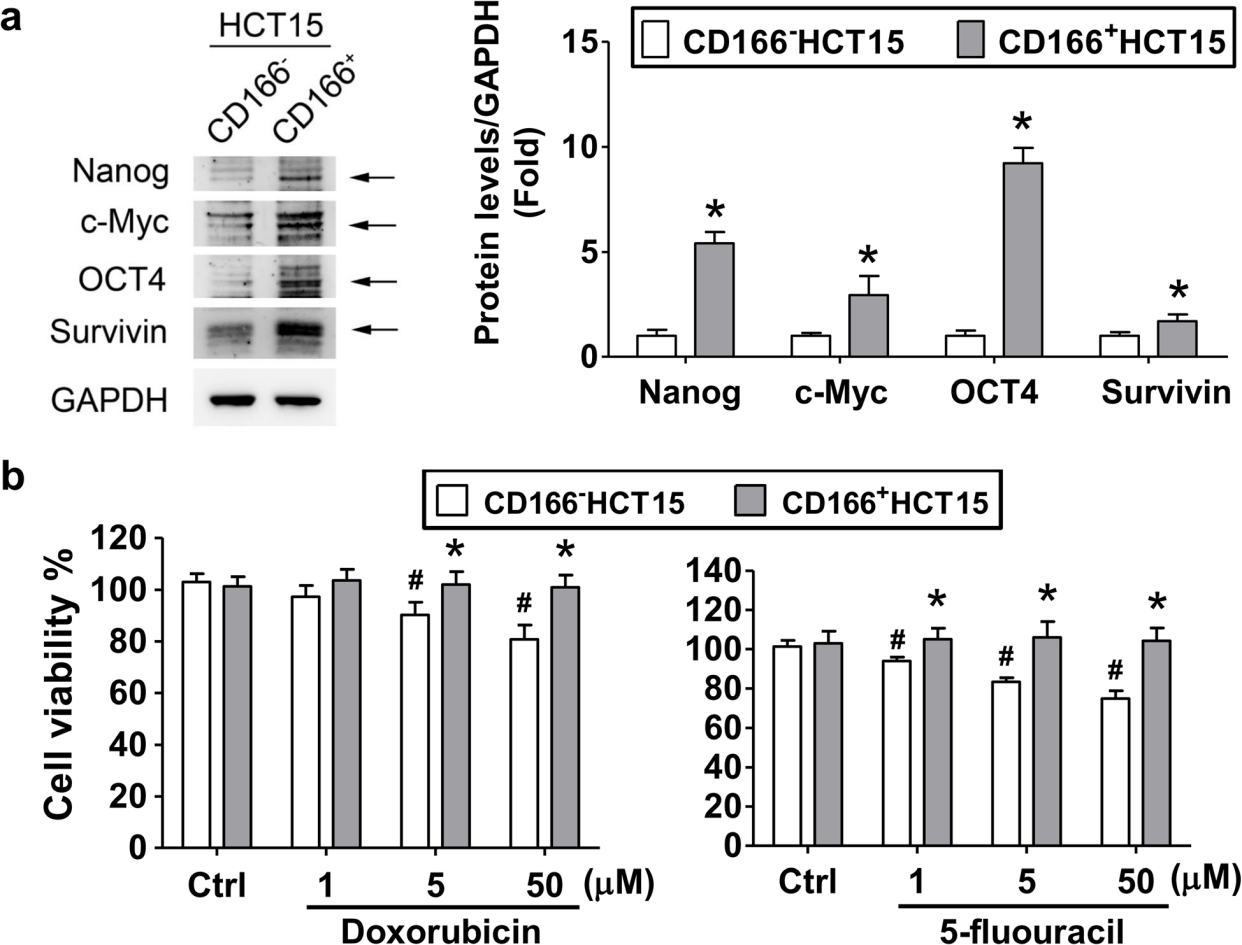
The protein expressions of cancer stem cell markers and drug resistance in CD166^+^HCT15 cells. **a** Four cancer stem cell markers Nanog, c-Myc, OCT4, and Survivin were detected by Western blotting and quantified by densitometry and normalized by GAPDH levels. The data are presented as mean ± SD (*n* ≥ 3). *P < 0.05, versus CD166^−^HCT15. The arrow indicated the protein position. **b** The effects of doxorubicin and 5-fluouracil on cell viability. Both CD166^+^HCT15 and CD166^−^HCT15 cells were treated with doxorubicin and 5-fluouracil (1–50 μM) for 24 h. Data are presented as mean ± SD (*n* ≥ 5). *P < 0.05, versus CD166^−^HCT15. ^#^*P* < 0.05, versus Ctrl (control)

In addition, several studies have found that the expressions of Nanog, c-Myc, OCT4, and survivin are linked to cancer stem cells [35–37]. In this study, we found that CD166^+^HCT15 cells showed significantly higher levels of Nanog, c-Myc, OCT4, and Survivin than that of CD166^−^HCT15 cells (Fig. 3a). We also found that CD166^+^HCT15 cells exhibited significant resistance to doxorubicin and 5-fluouracil as compared to CD166^−^HCT15 cells (Fig. 3b).

### Screening and identification of CD166-targeted peptides

**Fig. 4:**
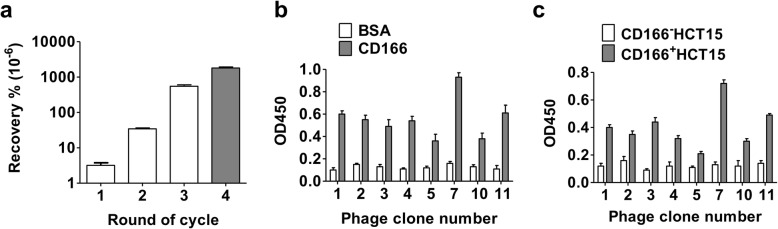
Screening and identification of CD166-targeted peptide. The phage display (12 phage library) was used to screen CD166 binding phages with four rounds of biopanning. **a** The titers of recovered phages from each round of biopanning were eluted by a blue/white colony screening on LB/IPTG/Xgal plate. The phage enrichment rate was calculated as output number/input number. **b** Phage ELISA assay. The carrying different peptide sequences of phage clones were exposed to CD166 protein and BSA (as a control) for detecting the binding affinity. **c** Phage binding to CD166 expression cells. The phage clones were further screened by cell-based phage ELISA. The CD166^+^HCT15 and CD166^−^HCT15 cells were positive and negative CD166 protein expression cells, respectively. Data are presented as mean ± SD (*n* ≥ 3)

In order to obtain the high-affinity target CD166 peptides, the phage display system was performed. The phage yields from each round of biopanning were used to indicate the enrichment of phages. The results showed that the fourth round of biopanning was highest for CD166 binding phages enrichment compared to third round of biopanning (Fig. 4a). The 30 phage clones were randomly picked from fourth round of biopanning for DNA sequencing, and obtained 8 corresponding amino acid sequences (P1–P8) (Table 1). To compare the binding capacity of these peptides to CD166 protein, the 8 phage clones (clone number 1, 2, 3, 4, 5, 7, 10, 11), which carried different peptide sequences, were picked and their binding affinities for CD166 were detected using phage ELISA and Cell-based phage ELISA. The results indicated that the one selection phage clone (clone number 7) showed highest binding to CD166 protein and CD166^+^HCT15 compared to other phage clones (Fig. 4b, c). Therefore, the P6 peptide (amino acid sequence: DSEGNSNLCSQS) was chemically synthesized for subsequent experiments.

### Effects of CD166-targeted peptides on binding ability in CD166^+^ CRC cells

**Fig. 5:**
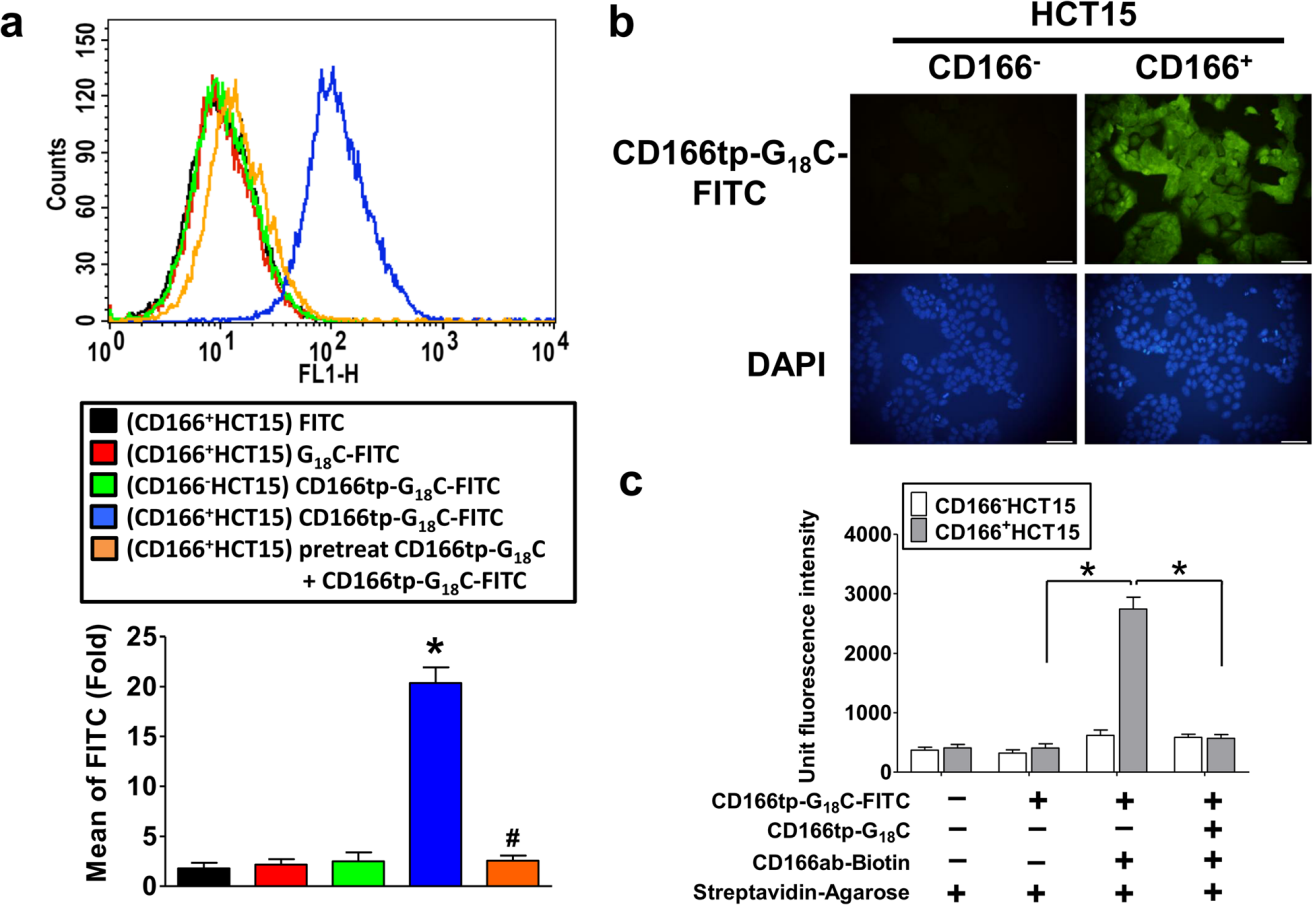
CD166tp-G_18_C targeted to CD166-positive CRC cells. **a** The CD166tp-G_18_C binding assay in CRC cells was analyzed by flow cytometry. Both CD166^+^HCT15 and CD166^−^HCT15 cells were treated with CD166tp-G_18_C-FITC (20 μg/mL) for 1 h. In competitive group, CD166^+^HCT15 cells were pre-treated with CD166tp-G_18_C (20 μg/mL) for 1 h and then treated with 20 μg/mL CD166tp-G_18_C-FITC for 1 h. Data are presented as mean ± SD (*n* ≥ 3). *P < 0.05, versus (CD166^−^HCT15) CD166tp-G_18_C-FITC; #*P* < 0.05, versus (CD166^+^HCT15) CD166tp-G_18_C-FITC. **b** The CD166tp-G18C binding assay in both CD166^+^HCT15 and CD166^−^HCT15 cells was analyzed by fluorescent microscopy. Both cells were treated with CD166tp-G_18_C-FITC (20 μg/mL) for 2 h. The total nuclei were stained with 4’, 6-diamino-2-phenylindole (DAPI). Magnification, × 200; scale bar, 100 μm. **c** CD166tp-G_18_C and CD166 protein interaction assay. Both CD166^+^HCT15 and CD166^−^HCT15 cells were treated with CD166tp-G_18_C-FITC (20 μg/mL) for 1 h. The supernatant of lysates was collected and treated with or without biotinylated-CD166 polyclonal antibody (2 μg/mL) in the presence of streptavidin agarose beads at 4 °C overnight. The fluorescent signaling of immunoprecipitates was analyzed by ELISA. The CD166tp-G_18_C (20 μg/mL) was pre-treated for competitive inhibition assay. Data are presented as mean ± SD (*n* ≥ 3). *P < 0.05

To provide chelating agent conjugation, reducing opsonization, and prolonging circulation time in vivo, the P6 peptide was extended an additional amino acid sequence (Glycine_18_-Cysteine, G_18_C). In order to understand the CD166 binding ability of CD166tp-G_18_C, the flow cytometric analysis was performed to detect the interaction between CD166tp-G_18_C and CD166^+^HCT15 cells. The fluorescence of CD166tp-G_18_C-FITC was significantly increased in CD166^+^HCT15 cells, but not in CD166^−^HCT15 cells (Fig. 5a). Pretreatment with non-fluorescence of CD166tp-G_18_C markedly decreased the binding efficiency of CD166tp-G_18_C-FITC in CD166^+^HCT15 cells (Fig. 5a). We further tested the binding ability of CD166tp-G_18_C-FITC in CD166^+^ and CD166^−^ HCT15 cells determined by immunofluorescence. The fluorescence imaging indicated that the binding ability of CD166tp-G_18_C-FITC was increased in CD166^+^HCT15 cell, but not in CD166^−^HCT15 cells (Fig. 5b). We further confirmed whether CD166tp-G_18_C-FITC targeted to the CD166 on the surface of CD166^+^HCT15 cells. The immunoprecipitation assay was performed with CD166 antibody. The results revealed that CD166tp-G_18_C-FITC could bind to CD166^+^HCT15 cells, but not CD166^−^HCT15 cells, via interacting with CD166 protein (Fig. 5c). The increased CD166tp-G_18_C-FITC binding could be inhibited by non-fluorescence CD166tp-G_18_C pretreatment.

### CD166 imaging in tumors and organs of CRC xenograft mice

**Fig. 6:**
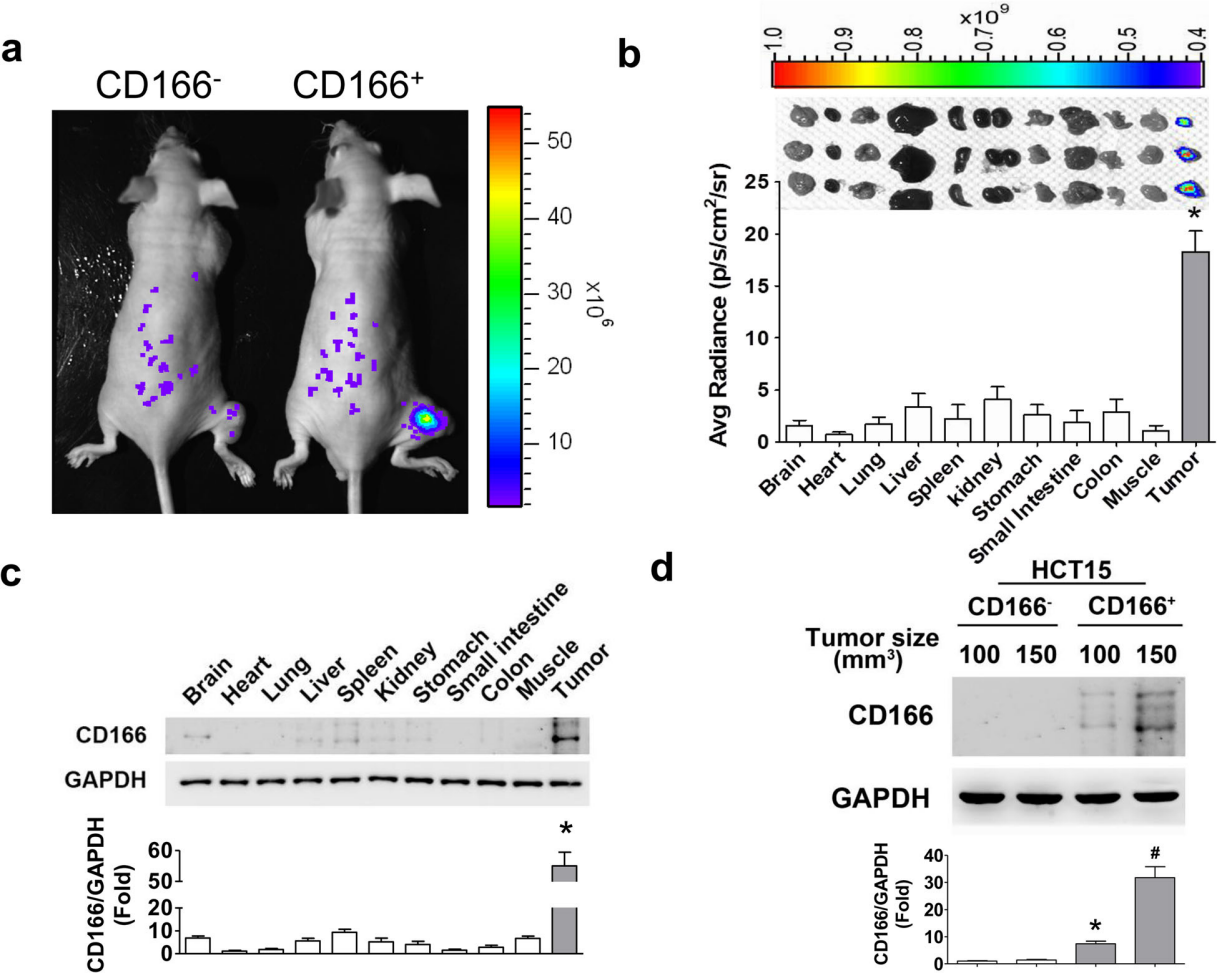
The CD166 imaging in tumors and organs of CRC xenograft mice. **a** The CD166 imaging of both CD166^+^HCT15 and CD166^−^HCT15 xenograft model. Both CD166^+^HCT15 and CD166^−^HCT15 cells were subcutaneously inoculated into the right flank of nude mice for establishing CD166 high- and non-expressed xenograft model, respectively. The Cy5.5-conjugated CD166tp-G_18_C was intravenously injected into both xenograft mice for 6 h and observed by in vivo imaging system. **b** The distribution of CD166 imaging in various organs and tumor tissue of CD166^+^HCT15 xenograft mice. The top panel presented the CD166 imaging in various organs. The button panel presented the quantitation of fluorescent signals. Data are presented as mean ± SD (*n* = 3). *P < 0.05, versus other organs. **c** The protein expressions of CD166 in tumors and various organs of CD166^+^HCT15 xenograft mice. **d** The CD166 expressions in tumors of both CD166^+^HCT15 and CD166^−^HCT15 xenograft mice. In **c** and **d**, protein expressions were determined by Western blotting and quantified by densitometry and normalized by GAPDH levels. In **c**, data are presented as mean ± SD (*n* = 3). *P < 0.05, versus other organs. In **d**, data are presented as mean ± SD (*n* = 3). *P < 0.05, versus 100 mm^3^ (CD166^−^HCT15); #*P* < 0.05, versus 150 mm^3^ (CD166^−^HCT15)

In order to assess whether CD166tp-G_18_C can be used as a diagnostic probe for CD166^+^ colorectal cancer (CRC) in vivo, the CD166^+^HCT15 and CD166^−^HCT15 xenograft mouse models were established. The fluorescence signaling was remarkably increased in the tumor area of CD166^+^HCT15 xenograft mice (Fig. 6a). However, weak fluorescence signaling was detected in CD166^−^HCT15 xenograft mice (Fig. 6a). We further detected the bio-distribution of CD166 in several organs of CD166^+^HCT15 xenograft mice. The fluorescence signaling was significantly and markedly increased in tumor tissue as compared to other organs in CD166^+^HCT15 xenograft mice (Fig. 6b). The expression of CD166 protein was also markedly upregulated in tumor tissue compared to other organs in CD166^+^HCT15 xenograft mice (Fig. 6c), which was consistent with the CD166 bio-distribution imaging. Moreover, the CD166 protein was significantly overexpressed in tumor tissue of CD166^+^HCT15 xenograft mice as compared to the CD166^−^HCT15 group (Fig. 6d).

### ^111^In-DTPA-CD166tp-G_18_C as a diagnostic radiopharmaceutical for CD166^+^ CRC tumor imaging detection in mice

**Fig. 7:**
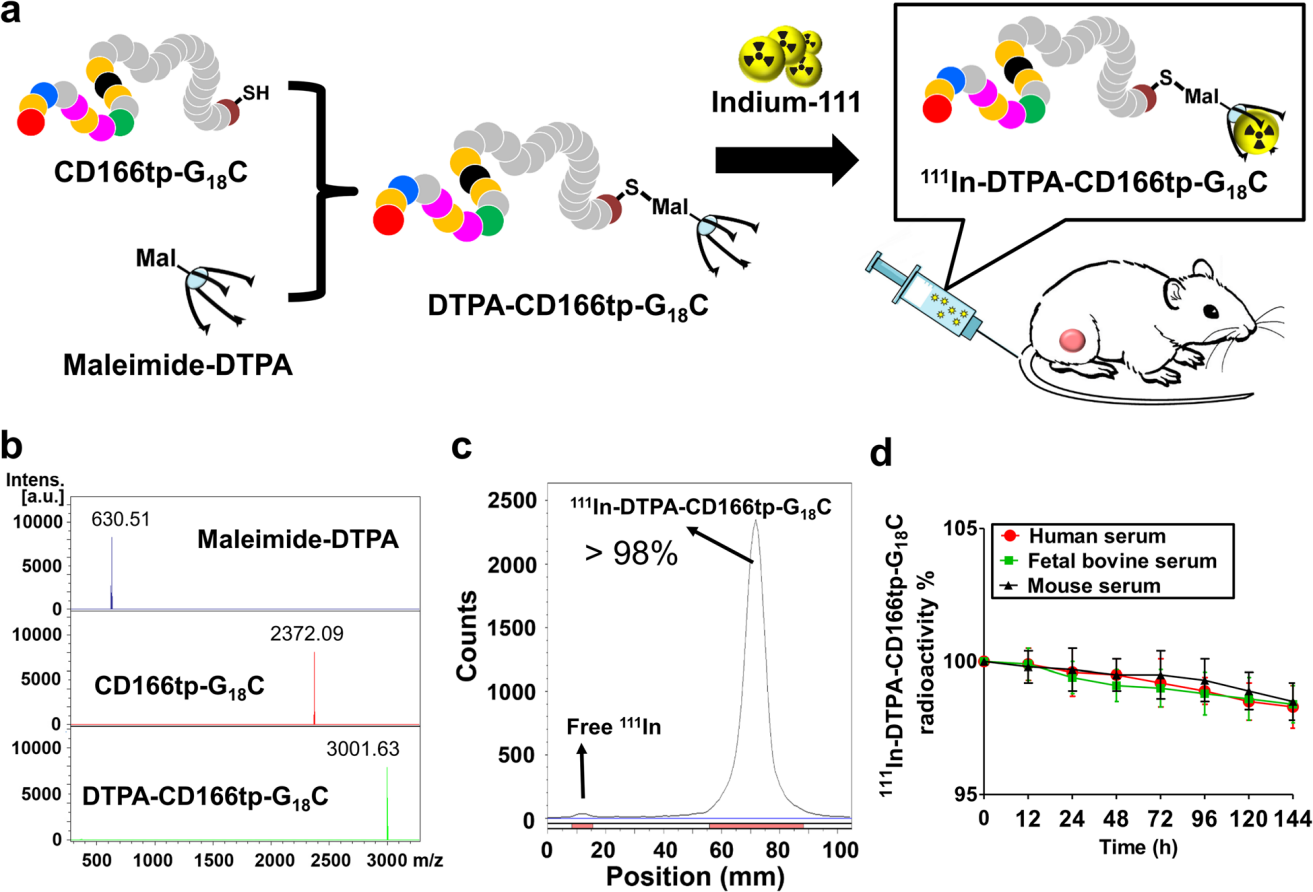
The synthesis and stability assay for ^111^In-DTPA-CD166tp-G_18_C. **a** The flowchart of ^111^In-DTPA-CD166tp-G_18_C synthesis. CD166tp-G_18_C was conjugated with DTPA for indium-111 labeling and then intravenously injected into CD166^+^HCT15 xenograft mice for CD166 detection in tumor area. **b** The molecular weights of DTPA-CD166tp-G_18_C detection. The molecular weights of Maleimide-DTPA, CD166-G_18_C, and DTPA-CD166tp-G_18_C were confirmed by a mass spectrometry. **c** The labeling efficiency of ^111^In-DTPA-CD166tp-G_18_C. After indium-111 labeling, instant thin-layer chromatography (ITLC) was performed to determine the radio-labeling efficiency in ^111^In-DTPA-CD166tp-G_18_C. **d** The stability of ^111^In-DTPA-CD166tp-G_18_C. The radioactivity of ^111^In-DTPA-CD166tp-G_18_C was detected in human, fetal bovine, and mouse serum for 144 h at 37 °C. The radioactivity was detected by ITLC. Data are presented as mean ± SD (*n* = 5)

In order to understand whether CD166tp-G_18_C can be used as a potential probe for CD166 detection in vivo, the radionuclide-labeled CD166tp-G_18_C was synthesized for detecting the CD166^+^ CRC in a xenograft mouse model. DTPA is one of the metal chelators for linking diagnostic probe and a corresponding radionuclides. Therefore, CD166tp-G_18_C was conjugated with DTPA for indium-111 labeling (^111^In-DTPA-CD166tp-G^18^C). The flowchart of ^111^In-DTPA-CD166tp-G_18_C synthesis and treatment method was shown in Fig. 7a. DTPA-CD166tp-G_18_C was successfully synthesized by conjugating the SH group of terminal cysteine of CD166tp-G18C with maleimide-DTPA. The molecular weight was determined by mass spectrometry (Fig. 7b). We further tested the labeling efficacy of Indium-111 onto the DTPA-CD166tp-G_18_C determined by instant thin-layer chromatography. The labeling efficacy of ^111^In-DTPA-CD166tp-G_18_C was more than 98% (Fig. 7c). Therefore, the molar activity at the end of labeling procedure was about 11.05 GBq/μmol. We further examined the stability of ^111^In-DTPA-CD166tp-G_18_C in sera of mouse, fetal bovine, and human during 144-h period. The result indicated that ^111^In-DTPA-CD166tp-G_18_C in these sera was more than 98% during 144 h period (Fig. 7d).

**Fig. 8:**
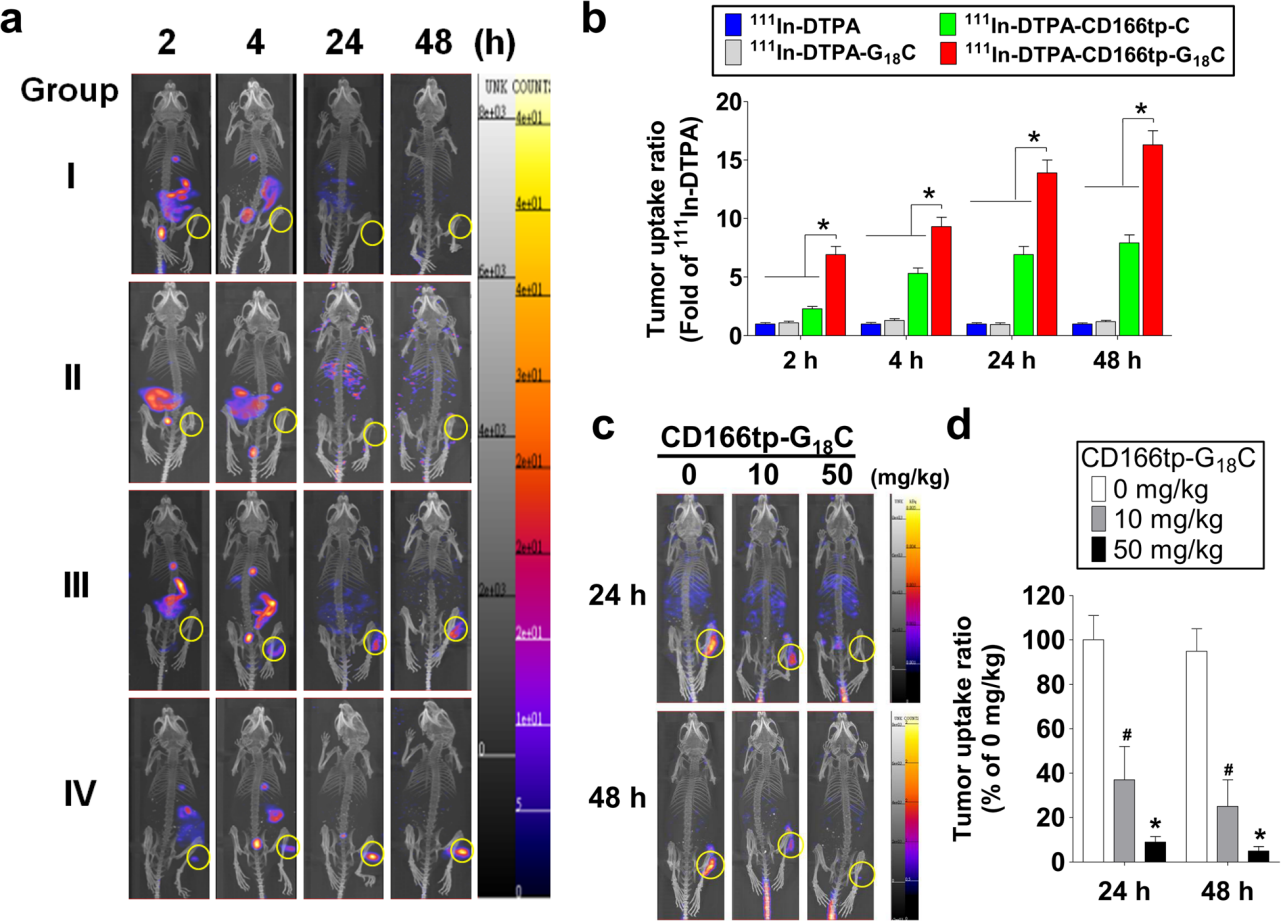
The nuclear imaging tracer of ^111^In-DTPA-CD166tp-G_18_C for detection of CD166-positive colorectal tumor in vivo. **a** The colorectal tumor nuclear imaging analysis in CD166^+^HCT15 xenograft mice. The ^111^In-DTPA-CD166tp-G_18_C and control groups (740 MBq/kg/per mouse) were intravenously injected into mice for 2, 4, 24, and 48 h and detected by a nanoSPECT/CT. Group I, ^111^In-DTPA; Group II, ^111^In-DTPA-G_18_C; Group III, ^111^In-DTPA-CD166tp-C; Group IV, ^111^In-DTPA-CD166tp-G_18_C. **b** Quantification of nuclear images in tumor areas of colorectal tumor xenograft mice. The circled positions in images were quantified by a 3D analysis software. Data are presented as mean ± SD (*n* ≥ 3). *P < 0.05, versus control group. **c** The competitive study of ^111^In-DTPA-CD166tp-G_18_C in CD166^+^HCT15 xenograft mice. After tumor xenograft mice were intravenously injected with CD166tp-G_18_C (0, 10, and 50 mg/kg) for 6 h, ^111^In-DTPA-CD166tp-G_18_C (740 MBq/kg/mouse) was intravenously injected into mice for 24 and 48 h and detected by a nanoSPECT/CT. **d** Quantification of nuclear images in tumor areas of colorectal tumor xenograft mice. Data are presented as mean ± SD (*n* ≥ 3). *P < 0.05, versus 0 mg/kg CD166tp-G_18_C group, #*P* < 0.05, versus 0 mg/kg CD166tp-G_18_C group

To investigate the targeting ability of ^111^In-DTPA-CD166tp-G_18_C in vivo, the CD166 imaging of CRC tumor was performed in CD166^+^HCT15 xenograft mice. After 2, 4, 24, and 48 h of injection, we observed that the level of accumulation of ^111^In-DTPA-CD166tp-G_18_C was significantly increased in the tumor as compared to other control groups (^111^In-DTPA, ^111^In-DTPA-G_18_C, and ^111^In-DTPA-CD166tp-C) (Fig. 8a, b). We also showed the competitive study of ^111^In-DTPA-CD166tp-G_18_C in CD166^+^HCT15 xenograft mice (Fig. 8c, d).

**Fig. 9:**
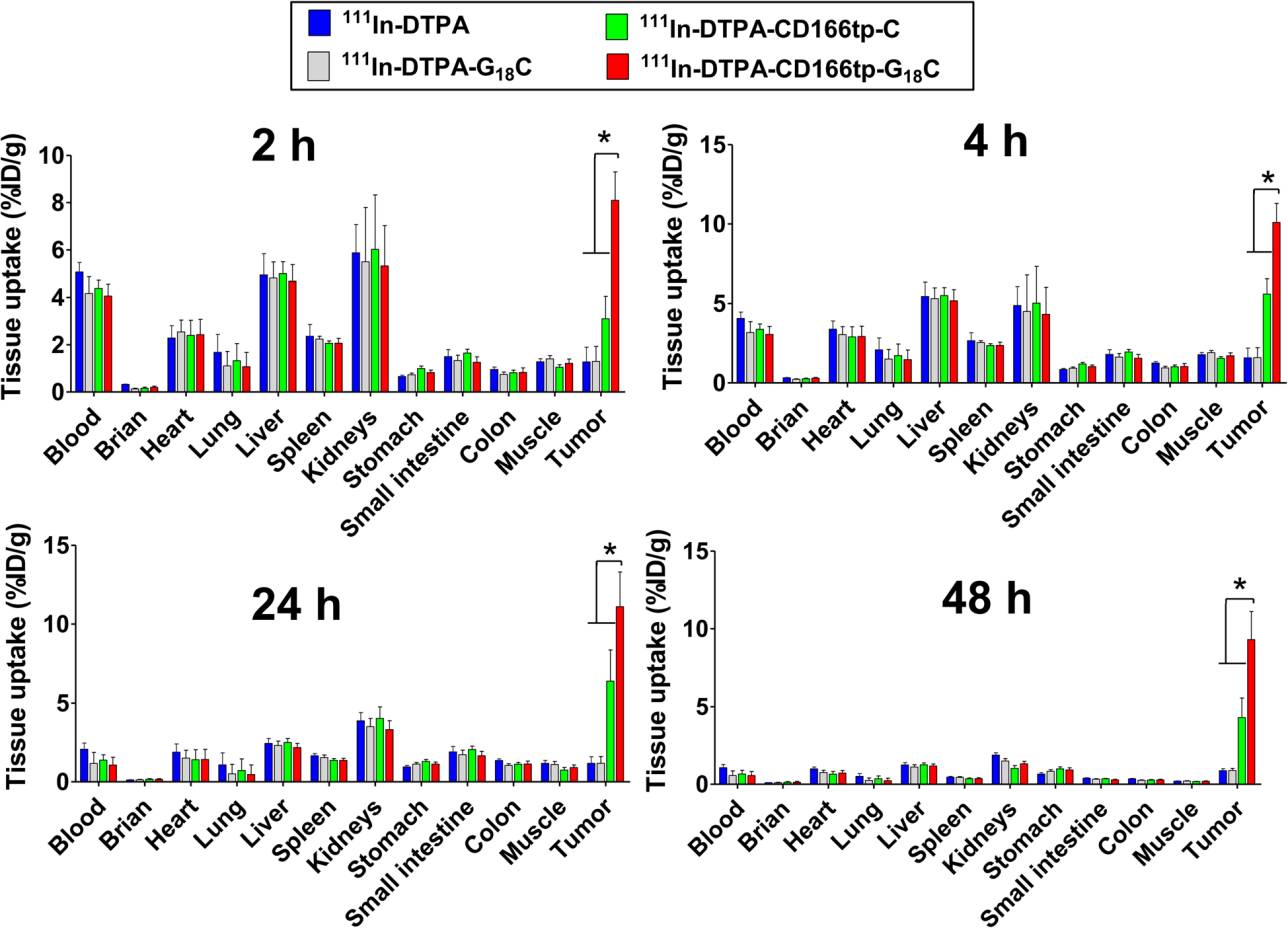
Tissue bio-distribution of ^111^In-DTPA-CD166tp-G_18_C in colorectal tumor xenograft mice. After ^111^In-DTPA-CD166tp-G_18_C and control groups (^111^In-DTPA, ^111^In-DTPA-G_18_C and ^111^In-DTPA-CD166tp-C) (148 MBq/kg/per mouse) intravenous injection for 2, 4, 24, and 48 h, CD166^+^HCT15 xenograft mice were sacrificed and obtained tumor, blood, and organs. The radioactivity of samples was detected by a gamma counter. Values are presented as the percentage of injected dose per gram organ (ID%/g). Data are presented as mean ± SD (*n* ≥ 3). *P < 0.05, versus control group

**Fig. 10:**
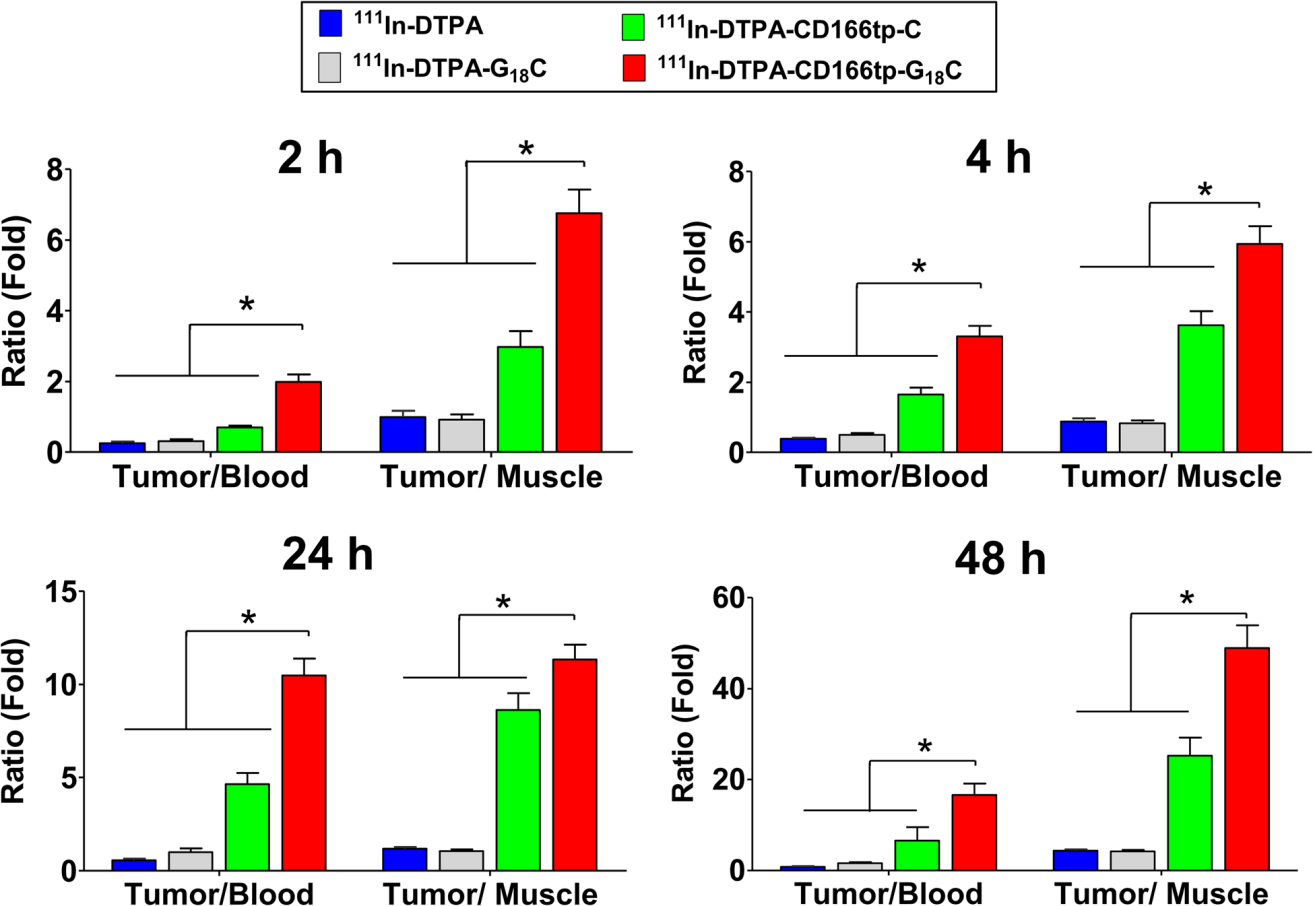
Tissue bio-distribution of ^111^In-DTPA-CD166tp-G_18_C in colorectal tumor xenograft mice. Tumor-to-muscle and tumor-to-blood ratios in ^111^In-DTPA, ^111^In-DTPA-G_18_C, ^111^In-DTPA-CD166tp-C, and ^111^In-DTPA-CD166tp-G_18_C treated-mice after 2, 4, 24, and 48 h injection were shown. Data are presented as mean ± SD (n ≥ 3). *P < 0.05, versus ^111^In-DTPA, ^111^In-DTPA-G_18_C and ^111^In-DTPA-CD166tp-C

To confirm the above results, the bio-distribution of ^111^In-DTPA-CD166tp-G_18_C was detected in tumor tissue and other organs of CD166^+^HCT15 xenograft mice. The results indicated that ^111^In-DTPA-CD166tp-G_18_C offered the highest level of radionuclide signal in the tumor tissue compared to radionuclide-labeled control compounds (^111^In-DTPA, ^111^In-DTPA-G_18_C, and ^111^In-DTPA-CD166tp-C) (Fig. 9). Moreover, the ratios of tumor-to-muscle (T/M) and tumor-to-blood (T/B) were evaluated in ^111^In-DTPA-, ^111^In-DTPA-G_18_C-, ^111^In-DTPA-CD166tp-C- or ^111^In-DTPA-CD166tp-G_18_C-treated CD166^+^HCT-15 xenograft mice. The ratios of T/M and T/B were remarkably increased in ^111^In-DTPA-CD166tp-G_18_C-treated group as compared to radionuclide-labeled control compounds (Fig. 10). These results confirmed the targeted efficiency of ^111^In-DTPA-CD166tp-G_18_C in CD166^+^ CRC tumor tissues.

## Discussion

In this study, we successfully identified CD166-positive CRC cells (CD166^+^HCT15) isolated from CRC cells and the characteristics of the cancer stem cells. We also screened the optimal CD166 targeting peptide (CD166tp) and added an additional amino acid sequence (G_18_C) to form a nuclear imaging probe (CD166tp-G_18_C). The CD166tp-G_18_C was confirmed to possess the ability of targeting the CD166-positive CRCs, which directly and specifically bound to CD166 on surface of CD166-positive CRCs. The fluorescent-labeled CD166tp-G_18_C was remarkably increased in the tumor of CD166^+^HCT15 xenograft mice compared to CD166^−^HCT15 xenograft mice. Moreover, we developed a nuclear imaging agent (^111^In-DTPA-CD166tp-G_18_C) using CD166tp-G_18_C as a probe for CD166-positive CRCs detection in a xenograft mouse model. In this xenograft model, when the tumor size achieved about 150 mm^3^ which possessed about 1 × 10^7^ CD166-postive cells (cancer cell average diameter: 15 μm), the nanoSPECT/CT detection started to perform. The difference of counts between background and tumor area was about 8 times. Therefore, we estimated that at least 1.25 × 10^6^ (or volume 18.75 mm^3^) CD166-postive cells were required for nanoSPECT/CT detection. These results suggest that ^111^In-DTPA-CD166tp-G_18_C can successfully target tumor site of CD166-positive CRCs for tumor imaging in vivo.

The present study identified a CD166 high-affinity target-binding peptide by using phage display system, which possessed the powerful targeted peptide screening ability [38]. The standard operating procedure recommended to perform 3 rounds of biopanning. Here, we performed 4 rounds of biopanning for confirming the credibility of CD166 affinity and reducing the number of peptides. The data of phage recovery rate indicated that the fourth round of biopanning was more than 3 times of CD166 binding phages enrichment compared to third round of biopanning (Fig. 4a). Therefore, if only 3 rounds were performed, 24 peptide sequences would be selected to cause more complicated screening operations and error rate. This concept was supported by previous studies [39, 40]. We have obtained a peptide sequence (P6) that has significantly greater affinity than other sequences (Fig. 4b, c) from assays of phage ELISA and cell-based phage ELISA. We further confirmed that the P6 peptide could effectively bind to CD166 protein with higher affinity in vitro and in vivo (Figs. 5 and 6). Moreover, Glycine (18)-Cysteine played the linking role between chelator and CD166 targeted probe. The uncharged Glycine is an ideal amino acid for extending peptide sequences. The sufficient length of linker is required for reducing the destruction of the spatial structure of the probe by the chelator and maintains high yields during peptide synthesis. We chose DTPA as a chelator, which not only could be labeled with Indium-111 for diagnosis, but also could be labeled with Lutetium-177 or Yttrium-90 for therapy [41].

The small population of cancer stem cells in tumor tissue is difficult to isolate from tumor tissue, which limits the ability to study the development and the pathogenesis of the cancer. In order to solve this problem, cell-based models of cancer stem-like cells were developed to extend our knowledge about CSCs, together with tumor biology, microenvironment, carcinogenesis, biomarker discovery, and improvement in oncologic therapies. CSCs can be obtained from tumor tissues or cancer cell lines through reprogramming [42, 43], expression of specific surface markers [44, 45], detection of the side population [46], selection of cells resistant to anoikis [47, 48], or culture in specific conditions [49]. Previous studies have confirmed that CD166 is a cell surface marker of CRCSCs and suitable for CRCSCs isolation [50, 51]. Therefore, we chose CD166 as a target of CRCSCs surface marker for CRCSCs isolation and identification in HCT15 cells. Fluorescence-activated cell sorting (FACS) and magnetic-activated cell sorting (MACS) are two commonly used methods. FACS is based on fluorochrome-conjugated antibodies in direct or indirect immune fluorescence staining. MACS is an alternative isolation method via super-paramagnetic and biodegradable microbeads coated with specific monoclonal antibodies that enrich a specific antigen cell type from a mixed population. However, FACS has some disadvantages such as high cost, low viability of recovered cells, and difficulty of using the complex equipment. Therefore, in this study, MACS was performed to collect high purity of CD166^+^ cells from HCT15 cells.

Some non-invasive molecular imaging technologies, such as optical techniques and nuclear imaging have been developed as detection tools for CSCs monitoring and tracking [52]. The macroscopic fluorescence and NIR imaging have been developed for CRCSC detection in vivo [53, 54]; however, the accumulation rate of agents was low and high background signaling was exhibited. Nuclear medicine imaging improved the limitation of tissue penetration in optical techniques and showed to be highly sensitive. In this study, we selected nuclear medicine imaging technique (single-photon emission computed tomography/computed tomography, SPECT/CT) and obtained the clear imaging and high accumulation rate more than 10 %ID/g at 4 h. Even though positron emission tomography/computed tomography (PET/CT) provides a much better sensitivity and spatial resolution than SPECT, the cost effectiveness and the greater availability of SPECT/CT scanners at most clinical centers contribute to more usage frequency of SPECT imaging than PET imaging [55].

Recently, the CD133-positive colorectal cancer stem-like cell was detected in vivo by using nanoSPECT/CT [56]. Liu et al. indicated that the radiolabeled CD133 targeted tracer (^99m^Tc-SHNH-AC133) showed high tumor uptake (8.82 ± 0.73%ID/g) and high tumor-to-muscle ratios (13.18 ± 2.84) at 36 h. Jin and colleagues have also developed the Iodine-125-labeled ANC9C5 as a radionuclide imaging agent for CSCs detection in colon carcinoma xenografts. But they obtained an unfavorable bio-distribution profile [57]. The CD166-targeted agent that we developed in this study showed higher tumor uptake (11.1 ± 2.2 %ID/g, 24 h) and tumor-to-muscle ratios (48.9 ± 5.03, 48 h) in CRCSCs xenograft. Compared to current CRCSCs nuclear imaging, we considered that the high specificity and good tumor targeting properties of ^111^In-DTPA-CD166tp-G_18_C may provide an optional imaging for tracking CRCSCs.

The CD166-targeted agent has been designed for imaging in vivo by using microPET [58]. Mccabe et al. indicated that the radiolabeled agent (^64^Cu-DOTA-CysDb) exhibited specific targeting of CD166-positive tumors in vivo at 4 h, but the high accumulation in the liver and kidney lead to low tumor uptake (2.4 ± 0.6 %ID/g) and low tumor-to-blood ratios (2.9 ± 0.6) in BxPC-3 xenograft model [58]. To increase the tumor-to-background ratio of the drug, Tavare et al. performed the reformatting CD166-targeted agents (^64^Cu-DOTA-3 L-amide-cDb and ^64^Cu-DOTA-3 L-thioether-cDb) on a CD166-overexpressed colorectal cancer (LS174T) xenograft model [50]. The bio-distribution assay confirmed that the LS174 tumor uptake was 1.4 ± 0.15%ID/g (ALCAM-3Lamide-DOTA-cDb) and 2.6 ± 0.53%ID/g (ALCAM-3 L-thioether-DOTA-cDb). They also found that the higher blood activity of both agents presented a slower blood clearance and a lower tumor-to-blood ratios (ALCAM-3Lamide-DOTA-cDb: 1.4, ALCAM-3 L-thioether-DOTA-cDb: 1.9) compared to previous agent (^64^Cu-DOTA-CysDb). In our study, the peptide-based agent (^111^In-DTPA-CD166tp-G_18_C) showed higher CD166-expressing tumor uptake (10.1 ± 1.26%ID/g, 4 h) and tumor-to-blood ratios (5.94 ± 0.51) compared to previous diabody-based agents. The difference in molecular weight between large and small molecular probes may be an important factor that leads to different imaging and bio-distribution.

To accurately carry the drug to the lesion area, many molecules with specific binding ability, such as antibodies, anti-peptides, and pharmacological inhibitors were developed as probes for targeting therapy and diagnosis. Demcizumab and vanticumab, the IgG2 monoclonal antibodies, blocked formation of active receptor signaling complex for CRCSCs therapy in clinical trials [59]. A FDA-approved drug Bevacizumab has been shown that it can target the vascular endothelial growth factor and possesses the potential against CRCSCs [60]. In addition, Gaedicke and colleagues have developed the antibody-based agent (^64^Cu-NOTA-AC133) for CD133^+^CSCs detection in subcutaneous and orthotopic glioma xenografts [61]. The antibody-based molecular probes were commonly used for cancer imaging, but the size limitation (~ 150 kDa) and immunogenicity made the antibodies no longer widely available for developing in vivo imaging [62]. In contrast, the peptide-based small-molecule probe that we developed in this study has the advantages of less immunogenicity, faster rates for tumor localization, convenient synthesis, and easier conjugation process. In the present study, comparing the ratio of tumor/blood between ^111^In-DTPA-CD166tp-C and ^111^In-DTPA-CD166tp-G_18_C (Fig. 10), we found that the CD166-targeted peptide with additional amino acid sequence improved the excretion rate (tumor/blood) of imaging agent in vivo and increased the bio-distribution in the tumor.

Di Stefano et al. have shown that the expression and localization of survivin, which plays the roles in cell death counteraction and mitotic progression control in CRCSCs, can be regulated by interleukin (IL)-4 [35]. The c-Myc-associated signaling has also been found to play the roles in CRCSCs regulation, chemotherapy resistance, and CRC organoids [36]. The induction of an embryonic stem cell-like transcriptional program consisting of specific reprogramming factors, such as Oct4, Nanog, Sox2, and KLF4, has been suggested to be involved in hypoxia-inducible factors (HIFs)-induced stem-like phenotype in cancer progenitors from multiple solid tumors during tumor hypoxia [37]. In the present study, we found that CD166^+^HCT15 cells showed significantly higher levels of Nanog, c-Myc, OCT4, and survivin than that of CD166^−^HCT15 cells, indicating that CD166^+^ cells have characteristics of CSCs.

## Conclusions

We demonstrated that CD166-positive CRC exhibited characteristics of CSCs, suggesting that it may be a useful drug screening tool for CRC diagnosis. The CD166tp-G_18_C compound specifically targeted to CD166-positive CRCs in vitro. We successfully synthesized DTPA-CD166tp-G_18_C and radiolabeled with Indium-111 for detecting CD166 imaging by using nanoSPECT/CT in CD166-positive CRC xenograft mice. The bio-distribution of ^111^In-DTPA-CD166tp-G_18_C confirmed the accumulation of CD166-positive cells in tumors. Therefore, ^111^In-DTPA-CD166tp-G_18_C may be a potential nuclear imaging agent for diagnosis of CRCSCs. The CD166 bound peptide-based nuclear imaging may provide physicians to classify cancer cells before treatment and monitor patients with a history of CRC after surgery or drug treatment.

## Data Availability

All data generated or analyzed during this study are included in this published article.

## Abbreviations

ADC: Antibody-drug conjugates
CD166^+^HCT15: CD166-positive HCT15 cells
CD166ab-FITC: FITC-conjugated CD166 antibody
CD166^−^HCT15: CD166-negative HCT15 cells
CD166tp: CD166-targeted peptides
CRC: Colorectal cancer
CRCSCs: Colorectal cancer stem cells
CSCs: Cancer stem cells
CT: Computed tomography
DTPA: Diethylenetriaminopentaacetic
G_18_C: Glycine18-Cysteine
ITLC: Instant thin-layer chromatography
MACS: Magnetic-activated cell sorting
PET: Positron emission tomography
SPECT: Single-photon emission computed tomography

## References

[1] Siegel RL, Miller KD, Jemal A (2018). Cancer statistics, 2018. CA Cancer J Clin..

[2] Fitzmaurice C, Allen C, Barber RM (2017). Global, Regional, and National Cancer Incidence, Mortality, Years of Life Lost, Years Lived With Disability, and Disability-Adjusted Life-years for 32 Cancer Groups, 1990 to 2015: A Systematic Analysis for the Global Burden of Disease Study. JAMA Oncol..

[3] Arnold M, Sierra MS, Laversanne M (2017). Global patterns and trends in colorectal cancer incidence and mortality. Gut..

[4] Moghimi-Dehkordi B, Safaee A (2012). An overview of colorectal cancer survival rates and prognosis in Asia. World J Gastrointest Oncol..

[5] Agliano A, Calvo A, Box C (2017). The challenge of targeting cancer stem cells to halt metastasis. Semin Cancer Biol..

[6] Ricci-Vitiani L, Lombardi DG, Pilozzi E (2007). Identification and expansion of human colon-cancer-initiating cells. Nature..

[7] Lu J, Ye X, Fan F (2013). Endothelial cells promote the colorectal cancer stem cell phenotype through a soluble form of Jagged-1. Cancer Cell..

[8] Medema JP (2017). Targeting the Colorectal Cancer Stem Cell. N Engl J Med..

[9] Guo M, Dou J (2015). Advances and perspectives of colorectal cancer stem cell vaccine. Biomed Pharmacother..

[10] Jones MF, Hara T, Francis P (2015). The CDX1-microRNA-215 axis regulates colorectal cancer stem cell differentiation. Proc Natl Acad Sci U S A..

[11] Zhai H, Fesler A, Ba Y, Wu S, Ju J (2015). Inhibition of colorectal cancer stem cell survival and invasive potential by hsa-miR-140-5p mediated suppression of Smad2 and autophagy. Oncotarget..

[12] Issa IA, Noureddine M (2017). Colorectal cancer screening: an updated review of the available options. World J Gastroenterol..

[13] Bowen MA, Patel DD, Li X (1995). Cloning, mapping, and characterization of activated leukocyte-cell adhesion molecule (ALCAM), a CD6 ligand. J Exp Med..

[14] Patel DD, Wee SF, Whichard LP (1995). Identification and characterization of a 100-kD ligand for CD6 on human thymic epithelial cells. J Exp Med..

[15] Van Kempen LC, Nelissen JM, Degen WG (2001). Molecular basis for the homophilic activated leukocyte cell adhesion molecule (ALCAM)-ALCAM interaction. J Biol Chem..

[16] Cayrol R, Wosik K, Berard JL (2008). Activated leukocyte cell adhesion molecule promotes leukocyte trafficking into the central nervous system. Nat Immunol..

[17] Ohneda O, Ohneda K, Arai F (2001). ALCAM (CD166): its role in hematopoietic and endothelial development. Blood..

[18] Tachezy M, Zander H, Gebauer F (2012). Activated leukocyte cell adhesion molecule (CD166)--its prognostic power for colorectal cancer patients. J Surg Res..

[19] Verma A, Shukla NK, Deo SV, Gupta SD, Ralhan R (2005). MEMD/ALCAM: a potential marker for tumor invasion and nodal metastasis in esophageal squamous cell carcinoma. Oncology..

[20] Klein WM, Wu BP, Zhao S (2007). Increased expression of stem cell markers in malignant melanoma. Mod Pathol..

[21] Ihnen M, Muller V, Wirtz RM (2008). Predictive impact of activated leukocyte cell adhesion molecule (ALCAM/CD166) in breast cancer. Breast Cancer Res Treat..

[22] Mezzanzanica D, Fabbi M, Bagnoli M (2008). Subcellular localization of activated leukocyte cell adhesion molecule is a molecular predictor of survival in ovarian carcinoma patients. Clin Cancer Res..

[23] Minner S, Kraetzig F, Tachezy M (2011). Low activated leukocyte cell adhesion molecule expression is associated with advanced tumor stage and early prostate-specific antigen relapse in prostate cancer. Hum Pathol..

[24] Dalerba P, Dylla SJ, Park IK (2007). Phenotypic characterization of human colorectal cancer stem cells. Proc Natl Acad Sci U S A..

[25] Vaiopoulos AG, Kostakis ID, Koutsilieris M, Papavassiliou AG (2012). Colorectal cancer stem cells. Stem Cells..

[26] Weichert W, Knosel T, Bellach J, Dietel M, Kristiansen G (2004). ALCAM/CD166 is overexpressed in colorectal carcinoma and correlates with shortened patient survival. J Clin Pathol..

[27] Sim SH, Kang MH, Kim YJ (2014). P21 and CD166 as predictive markers of poor response and outcome after fluorouracil-based chemoradiotherapy for the patients with rectal cancer. BMC Cancer..

[28] Beck A, Goetsch L, Dumontet C, Corvaia N (2017). Strategies and challenges for the next generation of antibody-drug conjugates. Nat Rev Drug Discov..

[29] Polakis P (2016). Antibody drug conjugates for cancer therapy. Pharmacol Rev..

[30] Hansel TT, Kropshofer H, Singer T, Mitchell JA, George AJ (2010). The safety and side effects of monoclonal antibodies. Nat Rev Drug Discov..

[31] David A (2017). Peptide ligand-modified nanomedicines for targeting cells at the tumor microenvironment. Adv Drug Deliv Rev..

[32] Wu CH, Liu IJ, Lu RM, Wu HC (2016). Advancement and applications of peptide phage display technology in biomedical science. J Biomed Sci.

[33] Wang Q, Li SB, Zhao YY (2018). Identification of a sodium pump Na(+)/K(+) ATPase alpha1-targeted peptide for PET imaging of breast cancer. J Control Release..

[34] Peng L, Shang W, Guo P (2018). Phage display-derived peptide-based dual-modality imaging probe for bladder cancer diagnosis and resection postinstillation: a preclinical study. Mol Cancer Ther..

[35] Di Stefano AB, Iovino F, Lombardo Y (2010). Survivin is regulated by interleukin-4 in colon cancer stem cells. J Cell Physiol..

[36] Elbadawy M, Usui T, Yamawaki H, Sasaki K. Emerging roles of C-Myc in cancer stem cell-related signaling and resistance to cancer chemotherapy: a potential therapeutic target against colorectal cancer. Int J Mol Sci. 2019;20. 10.3390/ijms20092340 PMC6539579 31083525

[37] Li Y, Laterra J (2012). Cancer stem cells: distinct entities or dynamically regulated phenotypes?. Cancer Res..

[38] Saw PE, Song EW (2019). Phage display screening of therapeutic peptide for cancer targeting and therapy. Protein Cell..

[39] Lingasamy P, Tobi A, Haugas M (2019). Bi-specific tenascin-C and fibronectin targeted peptide for solid tumor delivery. Biomaterials..

[40] Liu H, Zhao Z, Zhang L (2019). Discovery of low-molecular weight anti-PD-L1 peptides for cancer immunotherapy. J Immunother Cancer..

[41] Price EW, Orvig C (2014). Matching chelators to radiometals for radiopharmaceuticals. Chem Soc Rev..

[42] Sancho-Martinez I, Izpisua Belmonte JC (2016). Reprogramming strategies for the establishment of novel human cancer models. Cell Cycle..

[43] O'brien-Ball C, Biddle A (2017). Reprogramming to developmental plasticity in cancer stem cells. Dev Biol..

[44] Qureshi-Baig K, Ullmann P, Haan S, Letellier E (2017). Tumor-initiating cells: a criTICal review of isolation approaches and new challenges in targeting strategies. Mol Cancer..

[45] Tirino V, Desiderio V, Paino F (2013). Cancer stem cells in solid tumors: an overview and new approaches for their isolation and characterization. FASEB J..

[46] Shimoda M, Ota M, Okada Y (2018). Isolation of cancer stem cells by side population method. Methods Mol Biol..

[47] Harrison H, Farnie G, Howell SJ (2010). Regulation of breast cancer stem cell activity by signaling through the Notch4 receptor. Cancer Res..

[48] Shaker H, Harrison H, Clarke R (2017). Tissue Factor promotes breast cancer stem cell activity in vitro. Oncotarget..

[49] Calvet CY, Andre FM, Mir LM (2014). The culture of cancer cell lines as tumorspheres does not systematically result in cancer stem cell enrichment. PLoS One..

[50] Tavare R, Wu WH, Zettlitz KA (2014). Enhanced immunoPET of ALCAM-positive colorectal carcinoma using site-specific (6)(4)Cu-DOTA conjugation. Protein Eng Des Sel..

[51] Horst D, Kriegl L, Engel J, Kirchner T, Jung A (2009). Prognostic significance of the cancer stem cell markers CD133, CD44, and CD166 in colorectal cancer. Cancer Invest..

[52] Akbari-Birgani S, Paranjothy T, Zuse A (2016). Cancer stem cells, cancer-initiating cells and methods for their detection. Drug Discov Today..

[53] Zhang Z, Li M, Chen F (2016). Probe-based confocal laser endomicroscopy for imaging TRAIL-expressing mesenchymal stem cells to monitor colon xenograft tumors in vivo. PLoS One..

[54] Roy K, Kanwar RK, Kanwar JR (2015). LNA aptamer based multi-modal, Fe3O4-saturated lactoferrin (Fe3O4-bLf) nanocarriers for triple positive (EpCAM, CD133, CD44) colon tumor targeting and NIR. MRI and CT imaging. Biomaterials..

[55] Van Dort ME, Rehemtulla A, Ross BD (2008). PET and SPECT imaging of tumor biology: new approaches towards oncology drug discovery and development. Curr Comput Aided Drug Des..

[56] Liu Y, Jin X, Lan X (2019). SPECT imaging of colorectal cancer by targeting CD 133 receptor with 99mTc-labeled monoclonal antibody. Q J Nucl Med Mol Imaging..

[57] Jin ZH, Sogawa C, Furukawa T (2012). Basic studies on radioimmunotargeting of CD133-positive HCT116 cancer stem cells. Mol Imaging..

[58] Mccabe KE, Liu B, Marks JD (2012). An engineered cysteine-modified diabody for imaging activated leukocyte cell adhesion molecule (ALCAM)-positive tumors. Mol Imaging Biol..

[59] Garza-Trevino EN, Said-Fernandez SL, Martinez-Rodriguez HG (2015). Understanding the colon cancer stem cells and perspectives on treatment. Cancer Cell Int..

[60] Li CJ, Zhang X, Fan GW (2014). Updates in colorectal cancer stem cell research. J Cancer Res Ther..

[61] Gaedicke S, Braun F, Prasad S (2014). Noninvasive positron emission tomography and fluorescence imaging of CD133+ tumor stem cells. Proc Natl Acad Sci U S A..

[62] Khemthongcharoen N, Jolivot R, Rattanavarin S, Piyawattanametha W (2014). Advances in imaging probes and optical microendoscopic imaging techniques for early in vivo cancer assessment. Adv Drug Deliv Rev..

